# Endosonographic Findings and the Natural Course of Chronic Gastric Anisakiasis: A Single-Center Experience

**DOI:** 10.1155/2018/8562792

**Published:** 2018-09-20

**Authors:** Eun Young Park, Dong Hoon Baek, Gwang Ha Kim, Bong Eun Lee, So-Jeong Lee, Do Youn Park

**Affiliations:** ^1^Department of Internal Medicine, Pusan National University School of Medicine, and Biomedical Research Institute, Pusan National University Hospital, Busan, Republic of Korea; ^2^Department of Pathology, Pusan National University School of Medicine, Busan, Republic of Korea

## Abstract

**Background:**

Chronic gastric anisakiasis is a rare, usually asymptomatic, and difficult to diagnose infection incidentally discovered during endoscopy, resembling a subepithelial tumor (SET). Because its endoscopic ultrasonography (EUS) findings are not established, it is occasionally misdiagnosed as gastrointestinal mesenchymal tumors and removed by endoscopic or surgical resection. We aimed to assess the characteristic EUS findings of chronic gastric anisakiasis and the clinical course during follow-up.

**Methods:**

The database of all patients who underwent EUS at Pusan National University Hospital (Busan, Korea) between January 2011 and December 2016 was retrospectively analyzed. A total of 28 SET cases with EUS features suggesting chronic gastric anisakiasis were included in the study. The EUS, histopathologic, and follow-up endoscopic features were analyzed.

**Results:**

On EUS, the lesions were mainly located in the submucosal and/or propria muscle layers. Twenty-seven lesions (27/28, 96%) showed hypoechoic echogenicity, and 22 lesions (22/28, 79%) were heterogeneous. Hyperechoic tubular structures suggesting denaturalized Anisakidae larvae were seen in 22 lesions (22/28, 79%). Endoscopic biopsies revealed significant eosinophil infiltration (≥30 per high-power field) in 12 lesions (12/21, 57%). During the median follow-up period of 9 months (range, 1–55 months), SETs decreased or subsided in 26 lesions (26/28, 93%) with no change in the size of the two lesions (2/28, 7%).

**Conclusions:**

Chronic gastric anisakiasis, although rare, should be included in the differential diagnoses for gastric SETs, especially in regions where raw fish is widely consumed. EUS findings suggesting chronic gastric anisakiasis are heterogeneously hypoechoic lesions with hyperechoic tubular structures, mainly in the submucosal and/or muscularis propria layers. Because chronic gastric anisakiasis decreases or subsides in most cases, follow-up endoscopy 6–12 months later is recommended.

## 1. Introduction

Anisakiasis is a parasitic disease caused by an accidental ingestion of the nematode larva of the Anisakidae family in uncooked saltwater fish. This disease is caused by eating infected raw, pickled, or salted fishes such as herring, mackerel, squid, salmon, bonito, tuna, and cuttlefish. The incidence of gastric anisakiasis in a population is directly related to the consumption of raw fish. Therefore, the infection is prevalent in regions where raw fish is widely consumed, especially in Far East Asia, including Korea.

Gastrointestinal anisakiasis was first reported in 1937 [1], and it most commonly occurs in the stomach with an incidence of 68–75% [2, 3]. Most cases are acute gastric anisakiasis causing cramping abdominal pain, nausea, and vomiting. The diagnosis of acute gastric anisakiasis is usually by endoscopic confirmation, which often reveals the presence of the Anisakidae larvae or mucosal changes such as edema, erosion, ulceration, and hemorrhage.

However, chronic gastric anisakiasis, a kind of parasitic eosinophilic granuloma, is a rare entity; it is usually asymptomatic and difficult to diagnose because the Anisakidae larva is absent, and it often appears as an incidental subepithelial tumor (SET) during endoscopy. Because its endoscopic ultrasonography (EUS) findings are not yet established, it is sometimes misdiagnosed as gastrointestinal mesenchymal tumors or heterotopic pancreas and it is removed by endoscopic or surgical resection [4, 5]. Recently, there have been few reports on the EUS findings of chronic gastric anisakiasis presenting as a SET and its natural course. Therefore, the aim of this study was to assess the characteristic EUS findings of chronic gastric anisakiasis and its clinical course during follow-up.

## 2. Methods

**Figure 1:**
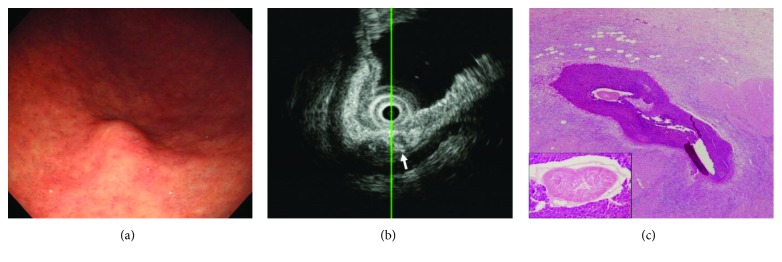
A case of chronic gastric anisakiasis histopathologically confirmed after surgical resection. (a) Endoscopy shows a subepithelial tumor-like lesion in the greater curvature of the gastric antrum. (b) On endoscopic ultrasonography, the lesion is a heterogeneously hypoechoic lesion in the submucosal layer. Hyperechoic tubular structures are seen inside the lesion (arrow). (c) Histopathological features of the resected specimen. Ill-defined granulomatous inflammation with marked eosinophil infiltration is seen in the submucosa (hematoxylin and eosin stain, ×40). Inside the granulomatous inflammation, the degenerated anisakiasis larva is observed (boxed area, hematoxylin and eosin stain, ×400).

The database of all patients who underwent EUS at Pusan National University Hospital (Busan, Korea) between January 2011 and December 2016 was retrospectively analyzed. Based on our previous cases of histologically confirmed chronic gastric anisakiasis (Figure 1), we identified 38 SET cases with EUS features suggesting chronic gastric anisakiasis. Of these, 10 cases that did not undergo follow-up endoscopy were excluded. Ultimately, a total of 28 SET cases with EUS features were included in this study. The study protocol was reviewed and approved by the Institutional Review Board at Pusan National University Hospital (H-1801-017-063).

### 2.1. Endoscopic Ultrasonography

EUS was performed using a radial scanning ultrasound endoscope (GF-UM2000; Olympus, Tokyo, Japan) at 7.5 and 12 MHz or a 20 MHz catheter probe (UM3D-DP20-25R; Olympus). All examinations were performed under intravenous conscious sedation (using midazolam with or without propofol). Scanning of the tumor was performed after filling the stomach with 300–600 mL of deaerated water. At least five still images were obtained for each lesion during EUS, and these images were saved in our database.

The EUS images were reviewed by a single experienced endosonographer (G. H. Kim) who had previously performed more than 1000 examinations. The following EUS features were analyzed: (a) location, (b) gross shape using the Yamada classification [6], (c) presence of mucosal erosion on endoscopy, (d) maximal diameter, (e) pattern of tumor growth (intraluminal, mural, or extraluminal), (f) endosonographic layer of origin, (g) echogenicity (hypoechoic, isoechoic, or hyperechoic), (h) homogeneity (homogenous or heterogeneous), (i) distinctness of the borders (distinct or indistinct), and (j) presence of hyperechoic tubular structures indicating the presence of denaturalized Anisakidae larvae.

### 2.2. Histopathological Evaluation

Hematoxylin and eosin slides were reviewed for cases in which endoscopic biopsy was performed, and the histological features (eosinophil count per high-power field (HPF)) were recorded. Significant eosinophilic infiltration was defined as when the number of eosinophils was ≥30 per HPF [7].

### 2.3. Statistical Analyses

Variables were expressed as medians or range and simple proportions. Statistical significance was evaluated using *χ*^2^ test or Fisher's exact test for categorical variables. A *P* value of <0.05 was considered statistically significant. The statistical analyses were conducted using IBM® SPSS® software, version 21.0 for Windows (IBM Corporation, Armonk, NY, USA).

## 3. Results

The 28 patients included 8 men and 20 women, age range from 25 to 76 years (median age: 53 years). Six patients presented with dyspepsia or epigastric pain. A SET was incidentally found during a routine health check-up in the other 22 patients who were asymptomatic. All patients except one had a history of marine raw fish intake within the previous 1 to 6 months.

**Figure 2:**
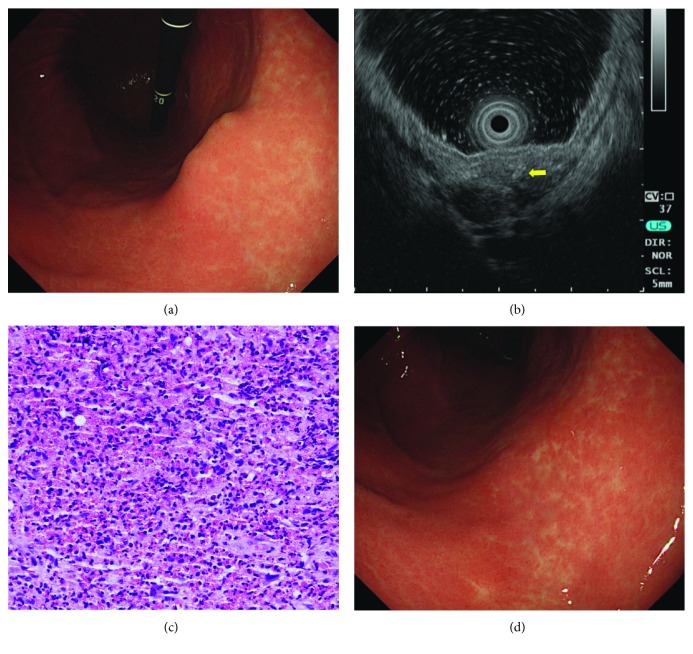
A representative case of chronic gastric anisakiasis (case 11). (a) Initial endoscopy shows a subepithelial tumor-like lesion in the lesser curvature of the gastric midbody. (b) On endoscopic ultrasonography, the lesion is a heterogeneously hypoechoic lesion in the submucosal and muscularis propria layers. Hyperechoic tubular structures are seen inside the lesion (arrow). (c) Endoscopic biopsy reveals increased eosinophil infiltration (hematoxylin and eosin stain, ×400). (d) Follow-up endoscopy performed 6 months later shows that the lesion has subsided completely.

Six lesions were located in the upper third of the stomach, 20 in the middle third, and one in the lower third (Table 1). Ten lesions (10/28, 35.7%) showed erosive change on the surface. As shown by the EUS, the lesions were mainly located in the third (submucosal) and/or fourth (propria muscle) layers and ranged from 3 mm to 25 mm in size (median size: 8 mm) (Table 2). A mural growth pattern was most commonly observed (23/28, 82%). Twenty-seven lesions (27/28, 96%) showed hypoechoic echogenicity, and 22 lesions (22/28, 79%) were heterogeneous. The borders were indistinct in 17 lesions (17/28, 61%), and hyperechoic tubular structures were seen in 22 lesions (22/28, 79%). Endoscopic biopsies using the bite-on-bite technique were performed in 21 lesions, and the mean count of eosinophils per HPF was 76 (range, 5–500). Significant eosinophil infiltration (≥30 per HPF) was seen in 12 lesions (12/21, 57%). A representative case (case 11) is shown in Figure 2.

During the median follow-up period of 9 months (range, 1–55 months), SETs decreased or subsided in 26 lesions (26/28, 93%) and there was no change in the size of two lesions (2/28, 7%). Of the 26 lesions which decreased or subsided, 16 lesions subsided completely during the median follow-up period of 8 months (range, 5–39 months). Among those with the presence or absence of hyperechoic tubular structures, all 22 lesions (100%) with hyperechoic tubular structures decreased or subsided, and only 4 of the 6 lesions (67%) without hyperechoic tubular structures decreased or subsided (*P* = 0.040). Of the 12 lesions with significant eosinophil infiltration, 10 lesions (83%) decreased or subsided, and all 9 lesions (100%) without significant eosinophil infiltration decreased or subsided (*P* = 0.486).

## 4. Discussion

Chronic gastric anisakiasis results from the invasion of the mucosal or submucosal layer by Anisakidae larvae, causing abscesses or eosinophilic granulomas; it can appear as a form of SET [3]. In the present study, characteristic EUS findings of chronic gastric anisakiasis were heterogeneously hypoechoic lesions with hyperechoic tubular structures, occurring mainly in the submucosal and/or muscularis propria layer. Most SET lesions decreased or subsided on the follow-up endoscopy. To our knowledge, this study is the first report of the EUS features of chronic gastric anisakiasis presenting as a SET and its natural course.

Anisakiasis is a zoonotic disease caused by an infection with the larvae of the nematode Anisakis, which migrates into the human viscera. The adult Anisakis lives in the stomach of marine mammals such as whales and dolphins. Crustaceans are the first intermediary hosts. The second intermediary hosts include various species of fishes and cuttlefishes. Humans are only accidentally contaminated [1, 8]. During the previous last 30 years, the number of reported gastrointestinal anisakiasis in the world literature is up to 13,000 with most cases reported in Korea and Japan, where raw fish is widely consumed. Favored fishes of Korean, such as mackerels, cods, Alaska pollacks, scabbard fish, and squids, are reported to be heavily infected with *Anisakis simplex* [9]. As a result, almost all the patients in the present study (27 patients) had a history of marine raw fish ingestion.

The clinical symptoms of gastric anisakiasis are classified as acute or chronic infections [10]. Acute anisakiasis infection is due to the invasion of the gastric wall by the larvae. The most common symptoms of acute gastric anisakiasis are severe epigastric pain, anorexia, and vomiting within 12 hours of raw fish ingestion [3]. Using endoscopy, the larvae can be found in 50% of patients with acute gastric anisakiasis [11], and mucosal edema, erythema, erosion, or ulceration can also be seen [12, 13]. Although the infection regresses gradually, it is sometimes misdiagnosed as a gastric ulcer or gastric cancer [14, 15]. Chronic anisakiasis infection is often difficult to diagnose because its symptoms are mild and nonspecific and the larvae are denaturalized and absorbed in the submucosal layer [16]. The diagnosis is often made incidentally during an endoscopy or after the discovery of a mass in the abdomen [17]. In the present study, only 6 patients (21%) had nonspecific symptoms such as dyspepsia or epigastric pain; the remaining 21 patients (79%) were asymptomatic.

Histologic findings of chronic anisakiasis are classified into four types according to the duration of infection and degree of larval denaturalization [3, 18]. The first type is the phlegmon type where larvae are located in the submucosal layer with eosinophil, neutrophil, and histiocyte infiltrations. The second type is the chronic abscess type; larvae are denaturalized, and an abscess is formed by eosinophils and fibrin. The third type is the abscess-granulomatous type. This type develops 6 months after Anisakidae larvae infection and shows progressive granuloma and fibrosis. The fourth type is the granulomatous type where the abscess becomes a granuloma. Considering these histologic findings arising mainly from the submucosal layer, chronic gastric anisakiasis appears as a SET-like morphology on endoscopy as shown in the present study.

EUS findings of acute gastric anisakiasis are thickening of the gastric wall, mainly of the submucosal layer with low echoic change [19]. However, there have been few reports of chronic gastric anisakiasis appearing as a SET and its EUS findings [5]. According to the present study, EUS findings that suggest chronic gastric anisakiasis are heterogeneously hypoechoic lesions with hyperechoic tubular structures, mainly in the submucosal and/or muscularis propria layer. These EUS findings are consistent with the aforementioned histologic findings of chronic anisakiasis: denaturalized larvae and abscess or granuloma formation in the submucosa. In particular, hyperechoic tubular structures are considered as indicative of the presence of a denaturalized larva. However, at a glance, these EUS findings are similar to those of gastric mesenchymal tumors, especially gastrointestinal stromal tumors [20, 21]. Therefore, some patients with chronic gastric anisakiasis undergo endoscopic or surgical resection to rule out the possibility of gastrointestinal stromal tumors [5, 22].

The role of endoscopic biopsy in chronic gastric anisakiasis is that endoscopic biopsy using the bite-on-bite technique enables us to obtain deep mucosal and submucosal tissues, which are the main pathologic sites of chronic anisakiasis. Thus, we could recognize the presence of eosinophils in all the lesions. However, eosinophils exist in the gastric mucosa of healthy persons or in some inflammatory conditions such as *Helicobacter pylori* gastritis and Crohn's disease. In a recent study involving the quantification of normal gastric eosinophil count, ≥30 eosinophils per HPF was suggested as the criteria of significantly increased eosinophils in gastric biopsies [7]. Based on these criteria, significant eosinophil infiltration was seen in 12 lesions (57%). The reason for this can be explained by the aforementioned histologic findings of chronic anisakiasis; as the time after the infection prolongs, the degree of eosinophil infiltration decreases.

Because chronic gastric anisakiasis is an inflammatory process, it is natural that the SET should decrease or subside. In addition, because the patients had a history of raw fish intake, characteristic EUS findings of chronic gastric anisakiasis, and significant eosinophil infiltration on biopsy, we decided to observe them rather than to perform endoscopic or surgical resection. As a result, most SETs (26/28, 93%) decreased or subsided; 16 lesions subsided completely during the median follow-up period of 8 months (range, 5–39 months). The median follow-up period for the 10 lesions, which decreased in size, was only 1 month (range, 1–55 months); in particular, the follow-up period for 9 lesions was less than 8 months. All lesions with hyperechoic tubular structures decreased or subsided. These results reveal that the lesions with hyperechoic tubular structures are in a relatively early state with the heavy inflammation of chronic anisakiasis compared to lesions without hyperechoic tubular structures.

This study had several limitations. First, there may have been potential selection or information biases resulting from the single-center retrospective nature of the study. Second, we did not confirm the Anisakidae larvae histopathologically. Immunologic methods using specific serum IgE antibody to *A. simplex* are reported to be helpful in the diagnosis of anisakiasis, but this antibody was detected in 25% of healthy controls and lacked specificity because of its cross-reactivity with other parasite antigens [23]. Furthermore, it is not generally available; thus, we could not utilize it in the present study. However, we experienced several cases of chronic gastric anisakiasis which was histopathologically confirmed by endoscopic or surgical resection, and then, we came to understand the EUS findings and corresponding histopathology of chronic anisakiasis. Finally, we did not perform endoscopic biopsies to evaluate the change in eosinophil counts on the follow-up endoscopy.

## 5. Conclusions

Although it is rare, chronic gastric anisakiasis should be included in the differential diagnoses for gastric SETs, especially in regions where raw fish is widely consumed. EUS findings suggesting chronic gastric anisakiasis include heterogeneously hypoechoic lesions with hyperechoic tubular structures, mainly in the submucosal and/or muscularis propria layers. In addition, endoscopic biopsy results showing significant eosinophil infiltration increase the possibility of chronic anisakiasis. Because chronic gastric anisakiasis decreases or subsides in most cases, follow-up endoscopy performed 6 to 12 months later is recommended.

## Figures and Tables

**Table 1:** Clinicopathologic and endosonographic features in 28 patients with chronic gastric anisakiasis.

Case	Sex	Age (years)	Location	Gross shape^∗^	Erosion	EUS features							Eosinophil no. on endoscopic biopsy^†^	Size on follow-up endoscopy	Follow-up period (months)
						Size (mm)	Growth pattern	Layer	Echogenicity	Homogeneity	Border	Hyperechoic tubular structures			
1	M	27	Middle	I	−	7	Mural	3	Hypoechoic	Heterogeneous	Distinct	+	N-P	Decreased	1
2	M	41	Upper	I	−	3	Mural	3	Hypoechoic	Heterogeneous	Distinct	+	41	Subsided	26
3	M	43	Middle	I	+	9	Mural	3	Hypoechoic	Heterogeneous	Indistinct	+	37	Decreased	1
4	M	49	Middle	I	+	25	Intraluminal	2	Hypoechoic	Homogeneous	Indistinct	−	22	Subsided	32
5	M	51	Middle	I	−	17	Mural	3	Hypoechoic	Heterogeneous	Indistinct	+	64	Subsided	9
6	M	53	Middle	I	−	5	Mural	3	Hypoechoic	Homogeneous	Indistinct	−	5	Decreased	5
7	M	58	Middle	I	−	10	Mural	3	Hypoechoic	Heterogeneous	Distinct	+	45	Subsided	9
8	M	71	Middle	I	+	7	Mural	2, 3	Hypoechoic	Heterogeneous	Indistinct	+	18	Subsided	9
9	F	25	Upper	I	−	7	Mural	3	Hypoechoic	Heterogeneous	Indistinct	−	30	Same	22
10	F	42	Lower	I	−	11	Mural	2	Hypoechoic	Homogeneous	Distinct	−	107	Same	13
11	F	45	Middle	I	+	17	Mural	3, 4	Hypoechoic	Heterogeneous	Indistinct	+	500	Subsided	6
12	F	47	Middle	I	−	6	Mural	3	Hypoechoic	Homogeneous	Distinct	+	12	Subsided	14
13	F	49	Middle	I	−	7	Intraluminal	3, 4	Hypoechoic	Heterogeneous	Indistinct	+	N-P	Subsided	12
14	F	49	Middle	II	+	15	Mural	3, 4	Hyperechoic	Heterogeneous	Indistinct	+	56	Subsided	8
15	F	52	Middle	I	+	7	Mural	3	Hypoechoic	Heterogeneous	Indistinct	+	15	Subsided	18
16	F	52	Middle	I	+	6	Mural	3	Hypoechoic	Heterogeneous	Distinct	+	20	Decreased	1
17	F	53	Upper	I	−	9	Intraluminal	3	Hypoechoic	Heterogeneous	Distinct	+	N-P	Subsided	53
18	F	53	Middle	I	−	11	Mural	3	Hypoechoic	Heterogeneous	Indistinct	+	73	Subsided	7
19	F	55	Middle	I	−	16	Mural	3	Hypoechoic	Heterogeneous	Distinct	+	90	Decreased	3
20	F	57	Upper	I	+	8	Mural	2, 3	Hypoechoic	Homogeneous	Indistinct	−	370	Subsided	1
21	F	59	Upper	I	+	4	Mural	3, 4	Hypoechoic	Heterogeneous	Distinct	+	N-P	Decreased	55
22	F	59	Middle	I	−	6	Mural	3	Hypoechoic	Heterogeneous	Indistinct	+	N-P	Subsided	11
23	F	62	Upper	I	−	7	Mural	3	Hypoechoic	Heterogeneous	Distinct	+	N-P	Decreased	1
24	F	64	Middle	I	−	12	Mural	3	Hypoechoic	Heterogeneous	Distinct	−	N-P	Decreased	1
25	F	66	Middle	I	−	11	Mural	3, 4	Hypoechoic	Heterogeneous	Indistinct	+	5	Decreased	1
26	F	66	Middle	I	−	13	Mural	3, 4	Hypoechoic	Heterogeneous	Indistinct	+	25	Subsided	15
27	F	75	Middle	I	−	8	Intraluminal	2	Hypoechoic	Homogeneous	Indistinct	+	55	Decreased	1
28	F	76	Upper	I	+	8	Intraluminal	3	Hypoechoic	Heterogeneous	Indistinct	+	11	Subsided	19

^∗^By Yamada classification [6]. ^†^Per high-power field. N-P: not performed.

**Table 2:** Summary of endosonographic features of chronic anisakiasis.

EUS features	*N* = 28 (%)
Median size, mm (range)	8 (3–25)
Growth pattern	
Intraluminal	5 (18)
Mural	23 (82)
Layer	
Second layer	3 (11)
Second and third layers	2 (7)
Third layer	17 (61)
Third and fourth layers	6 (21)
Echogenicity	
Hypoechoic	27 (96)
Hyperechoic	1 (4)
Homogeneity	
Homogenous	6 (21)
Heterogeneous	22 (79)
Border	
Indistinct	17 (61)
Distinct	11 (39)
Hyperechoic tubular structure	
Present	22 (79)
Absent	6 (21)

## Data Availability

The data used to support the findings of this study are available from the corresponding author upon request.
